# Liver Transplantation in Patients with Portal Vein Thrombosis: Comparing Pre-MELD and MELD era

**Published:** 2012-08-01

**Authors:** R. F. Saidi, N. Jabbour, Y. F. Li, S. A. Shah, A. Bozorgzadeh

**Affiliations:** *Division of Organ Transplantation, Department of Surgery, University of Massachusetts Medical School, Worcester, MA, USA*

**Keywords:** Liver transplantation, Portal vein thrombosis, MELD, Prognosis

## Abstract

Background: Portal vein thrombosis (PVT) used to be a relative contraindication for liver transplantation (LT). This obstacle has been dealt with following the improvement of LT-related techniques.

Objective: To compare the outcome of adult patients with PVT who underwent LT before and after adopting MELD.

Methods: We retrospectively searched our database for deceased donor LT recipients who had PVT, were operated between 1990 and 2009, and were 18 years old or more. The outcome of patients operated in pre-MELD era (1990–2001) was then compared with that of those operated in MELD era (2002–2009).

Results: The incidence of patients undergoing LT with PVT has increased from 1.2% (491/40,730) in pre-MELD era to 6% (2540/42,601) in MELD era (p<0.01). Patients with PVT in MELD era were older (53.6 *vs* 50.5), had higher calculated MELD (21.3 *vs* 18.9), shorter length of hospital stay after LT (25 *vs* 21.7 days), more likely to develop HCC (14.8% *vs* 0), and more likely to receive DCD allograft (3.9% *vs* 0.8%). Donor risk indices were comparable in both groups (1.9 *vs* 1.9). The median waiting time before transplantation decreased during MELD era (71 *vs* 99 days). Allograft and patients survival was comparable between the two eras. However, allograft and patients survival rates were lower in patients with PVT compared to those without. In Cox regression analysis, PVT was associated with worse allograft (HR=1.3, 95% CI: 1.2–1.4, p<0.001) and patient survival (HR=1.3, 95% CI: 1.2–1.5, p<0.001) compared to non-PVT patients.

Conclusions: The incidence of patients with PVT has increased in MELD era without improvement in outcomes. Donor and recipients characteristics changed in MELD era. PVT is still associated with poor outcomes compared to patients without PVT.

## INTRODUCTION

Portal vein thrombosis (PVT) is a common complication of chronic liver disease with an incidence that varies between 0.6% and 15.8% [1-4]. In the initial era of liver transplantation (LT), PVT was considered as an absolute contraindication for the procedure. However, innovations in surgical techniques and the use of aggressive approaches have made it possible to overcome PVT during LT, which is currently the only way to cure patients with end-stage liver disease and concurrent PVT. Nonetheless, the preoperative condition and extensive collateral circulation of these patients render LT complicated, and the complexity of the involved surgical techniques remains a challenge for transplant surgeons. The most important issue is the reconstruction of the portal system, for which several available surgical techniques have been proposed to ensure restoration of adequate portal flow during LT [5, 6]. In this study, we retrospectively reviewed Scientific Registry of Transplant Recipients (SRTR) data and compared the outcome of LT in patients with PVT in pre-MELD *vs* MELD eras.

## PATIENTS AND METHODS

We retrospectively queried the SRTR database for deceased donor LT recipients who had PVT, were operated between 1990 and 2009, and were 18 years old or more; we excluded partial and multiple LTs (n=3031). The cohort was then stratified into pre-MELD (1990–2001) and MELD eras (2002–2009).

In the SRTR database, PVT status is reported at two different times. It is reported for LT candidates (recorded as of the time of listing) and for transplant recipients (recorded as of the time of transplant). For analysis involving transplant recipients, the PVT field from the recipient records was used. On occasion, the PVT fields in the candidate and recipient files did not correlate (2.0% of patients). We did not specifically make adjustments when the two PVT covariates were not in agreement.

χ^2^ and *Student’s t* tests were used for comparison of proportions and means, respectively. Allograft and patient survival rates were the primary outcomes measured. Kaplan-Meier survival analysis was used for allograft and patient survival estimates. Variables with more that 20% missing values were excluded from the analysis. We originally included the following factors for unadjusted analysis: recipient age and sex, donor age and sex, diagnosis, MELD, length of hospital stay, and race. An unadjusted comparison of survival rate was performed using the log-rank test. Hazard ratios (HR) were estimated using Cox proportional-hazard methodology and estimates are reported as HR (95% confidence interval [CI]). Multivariate Cox modeling was performed using potential risk factors and covariates that were found to be statistically significant in unadjusted Cox models. Statistical significance was defined as p<0.05.

This study was reviewed by the University of Massachusetts Medical School Institutional Review Board (IRB) and deemed appropriate for exemption from IRB oversight as no personal identifiers were used among datasets. 

## RESULTS

There were 83,331 adult patients who underwent deceased donor LT from 1990 to 2009. The cohort was then stratified into pre-MELD (n=40,730) and MELD era (n=42,601). The incidence of LT for PVT increased from 1.2% (n=491) in pre-MELD era to 6% (n=2540) in MELD era (p<0.01). Table 1 shows characteristics of the two groups. The age of recipients with PVT increased in MELD era. There was no significant difference between the two groups in terms of recipient gender, race, and donor age and gender. The median waiting time from listing to transplantation decreased in MELD era. The length of hospital stay (25 *vs* 21.7 days) and retransplantation rate (17.3% *vs* 12.7%) decreased in the MELD era. Interestingly, the number of patients with hepatocellular carcinoma (HCC) and PVT increased from 0 in pre-MELD era to 294 (14.8%) in MELD era. Although the DRI of allograft was the same in both eras (1.9), the utilization DCD allograft increased.

**Table 1:** Donors and recipients characteristics of patients with portal vein thrombosis who underwent liver transplantation in pre-MELD and MELD era

Variable	pre-MELD	MELD	p value
	491/40,730 (1.2%)	2540/42,601 (6%)	
Recipient age	50.5±10.1	53.6±9.3	<0.001
Recipient gender: male	338 (69%)	1574 (62%)	0.2
Recipient race			0.4
White	444 (91%)	2236(88%)	
Black	24 (5%)	203 (8%)	
Other	23 (4%)	101 (4%)	
Donor age	38.6±16.7	39.8±17.2	0.1
Donor gender: male	280 (57%)	1397 (55%)	0.9
Wait-time (median)	99	71	<0.001
CIT	8.3±3.4	7.5±3.4	0.2
LOS	25±32	21.7±28	<0.001
RE-TX	85 (17.3%)	253 (12.7%)	0.01
MELD	18.9±8	21.3±8.5	<0.001
DRI	1.9±0.4	1.9±0.4	0.1
HCC	294 (14.8%)	0	
SPLIT	472 (3.8%)	104 (4.1%)	0.07
DCD	4 (0.8%)	73 (3.8%)	0.001

**Figure 1:**
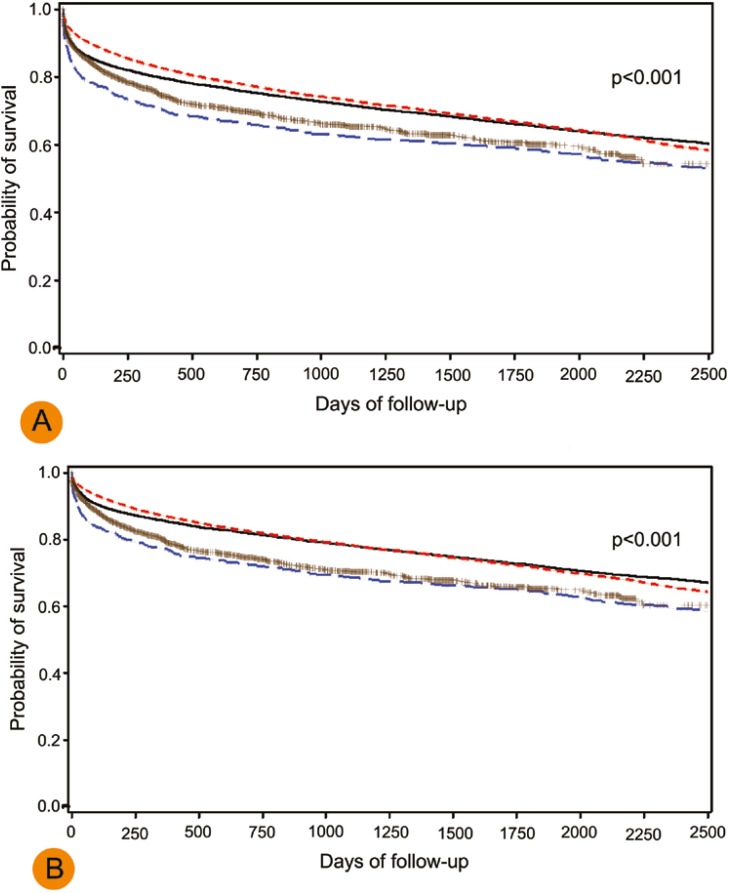
Allograft (A) and patients (B) survival of liver transplant recipients with and without portal vein thrombosis who operated in pre-MELD and MELD era. Black: No PVT (1990–2001); Red: No PVT (2002–2009), Blue: PVT (1990–2001); Purple: PVT (2002–2009),

Overall, patients with PVT had worse allograft and patient survival rates in both eras when compared with patients without PVT (Fig 1). However, the rates were comparable in patients with PVT in both eras. In Cox regression analysis, PVT was associated with worse allograft (HR=1.3, 95% CI: 1.2–1.4, p<0.001) and patient survival (HR=1.3, 95% CI: 1.2–1.5, p<0.001) compared to non-PVT patients.

**Table 2:** Cox proportional hazard model predicting allograft (A) and patient survival (B)

A		
Variables	HR (95% CI)	p value
Age	1.01 (1.01–1.03)	0.003
Sex (female)	1.24(101–1.54 )	0.038
PVT	1.2 (1.2–1.4)	0.03
MELD	1.1 (0.89–1.23)	0.1
BMI	1.01(0.99–1.04)	0.07
Donor age	1.02 (1.01–1.03)	<0.001
Donor sex (female)	0.88 (0.71–1.07)	0.2
DRI	1.39 (1.27–1.55)	<0.001
B.		
Variables	HR (95% CI)	p Value
Age	1.01 (1.01–1.04)	<0.001
Sex (female)	1.23 (1.03–1.46)	0.01
PVT	1.3 (1.2–1.5)	<0.001
MELD	1.12 (0.98–1.45)	0.25
BMI	1.08 (0.95–1.1)	0.09
Donor age	1.0 (0.99–1.05)	0.06
Donor sex (female)	0.78 (0.68–1.11)	0.3
DRI	1.44 (1.35–1.53)	<0.001

## DISCUSSION

Although PVT was considered an absolute contraindication for LT in the early 1980’s, advances in surgical techniques and perioperative management have overcome this obstacle. Several groups have reported favorable results in patients with PVT undergoing LT, and have described effective strategies for the management of PVT during LT [6-13]. PVT is therefore, no longer a contraindication for LT. Several single center studies have reported the incidence of PVT during LT from 2.1% to 26% [10-16]. Some studies show that the long-term outcome of patients with PVT undergoing LT is comparable with that of patients without PVT [8-12]. Our study showed that some outcomes improved in MELD era, such as length of hospital stay and retransplantation rate. However, the overall allograft and patient’s survival rates remained unchanged despite the fact that donors and recipients characteristics changed significantly.

In this study, we showed that the incidence of LT for PVT increased from 1.2% (n=491) in pre-MELD era to 6% (n=2,540) in MELD era (p<0.01). Comparing PVT patients who underwent LT in pre-MELD *vs* MELD era, we found that the recipients were older, had higher MELD score, and were more likely to have HCC. DRI was comparable in both groups. The utilization DCD allograft increased in the MELD era. The median waiting time also decreased in the MELD era.

Our study showed that PVT is a risk factor for poor allograft and patient outcomes. Englesbe also showed that compared with LT candidates without PVT, those with PVT do not have different rates of LT or survival on the waiting list [17]. In contrast, LT recipients with PVT have significantly inferior survival. The reason for the differences in outcome is not completely explained by the data available for the present analysis; this warrants further large-scale investigations as well. Interestingly, 14.8% of patients with PVT in MELD era had HCC. The occurrence of malignant thrombosis in cirrhotic patients concurrent with HCC is possible. Therefore, if patients with HCC are considered for LT, it is advisable to determine with certainty which PVTs are not malignant thromboses. 

Another concern in transplantation in patients with PVT is postoperative PV rethrombosis. It has been reported that 6.2% to 28.6% of patients experience PV rethrombosis after LT [18-20]. Severe rethrombosis can also lead to a high incidence of mortality. Utilization of anticoagulation therapy to prevent PV rethromobosis after LT remains controversial, but it could be considered in high risk patients or for recanalization of PVT after LT [22]. Taken together, depending on PVT grading and the experience of the surgeon, various surgical techniques can be performed to restore adequate portal flow to liver grafts. Briefly, a thrombectomy with direct PV anastomosis is indicated in cases presenting with a mild degree of PVT. A jump venous graft from the SMV is only indicated for the restoration of portal flow in cases of extensive PVT, and if there is no suitable engorged collateral CV available. Moreover, to ensure successful transplantation in patients with PVT, preoperative evaluation and thorough planning, as well as the ideal management of thrombus during LT, are essential.

This study has several limitations. First, it is a retrospective analysis of SRTR data. We recognize both potential advantages and limitations of this study that uses a large national database. However, the large sample size provides sufficient power to detect significant independent risk factors that may be missed by single-center studies. As with any analysis utilizing the SRTR database, our conclusions rely on the assumption that there is no systematic bias generated by reporting error or missing data. However, the primary endpoint for this analysis was allograft and patient survival, which is reliably captured in the SRTR database. Residual or unmeasured confounders that could impact allograft and patient survival include: surgeon technique, differences in immunosuppression protocols, the fat content/quality of the allograft and center-specific practices. Second, we were not able to analyze center-specific outcomes.

In summary, our study showed that the incidence of patients with PVT who undergo LT has increased in MELD era.

## Notes

**FINANCIAL SUPPORT:** None declared.
